# Pineal gland volume loss in females with multiple sclerosis

**DOI:** 10.3389/fnana.2024.1386295

**Published:** 2024-05-15

**Authors:** Miloš Vuković, Igor Nosek, Jasmina Boban, Duško Kozić

**Affiliations:** ^1^Department of Radiology, Faculty of Medicine Novi Sad, University of Novi Sad, Novi Sad, Serbia; ^2^Department for Radiology Diagnostics, Oncology Institute of Vojvodina, Sremska Kamenica, Serbia

**Keywords:** demyelination, disease, myelin, melatonin, sunlight, vitamin D

## Abstract

**Introduction:**

Multiple sclerosis has a complex pathophysiology, and numerous risk factors can contribute to its development, like exposure to sunlight that is associated with serum levels of melatonin. The aim of this study was to determine whether the volume of the pineal gland, assessed by magnetic resonance imaging (MRI), correlated with the presence of multiple sclerosis.

**Methods:**

This retrospective study included a total of 394 patients. Subjects were divided into two groups: the first group consisted of 188 patients with a definite diagnosis of multiple sclerosis (based on revised McDonald criteria) and the second group consisted of 206 healthy controls. To examine the influence of age on pineal gland volume, we stratified the whole sample into three age groups: first involved patients under 20 years, second patients between 20 and 40 years, and third group included patients over 40 years. The maximum length (L) and height (H) of the pineal gland were measured on the T1-weighted sagittal images, and the width (W) was measured on the T2-weighted coronal or axial images. The volume of the gland was calculated as an approximation to an ellipse, according to the formula *V* = (*L* × *H* × *W*)/2.

**Results:**

Pineal gland volume of female multiple sclerosis (MS) patients (*N* = 129) was significantly lower than in healthy females (*N* = 123) (*p* = 0.013; *p* < 0.05), unlike in males where there is not such difference. Also, pineal gland volume is not age-dependent, and the observed smaller pineal gland in MS patients can reliably be attributed to the disease itself. Additionally, large pineal gland size, especially over 62.83 mm^3^ when compared to pineal gland volume below 31.85 mm^3^ is associated with more than double reduced risk of multiple sclerosis (OR 0.42; *p* = 0.003).

**Discussion:**

Our results suggest that women with multiple sclerosis have smaller pineal glands that can theoretically be explained by a lack of input stimuli and the resultant decrease in gland volume. Additionally, the risk of multiple sclerosis is reduced in larger pineal gland volumes.

## 1 Introduction

Multiple sclerosis (MS) is a central nervous system inflammatory demyelinating disease, which is the leading cause of nontraumatic neurologic disability in young adults and is estimated to affect around 2.3 million people globally (Browne et al., 2014). Although the pathogenesis of MS is yet to be fully understood (Wootla et al., 2012), studies have shown that the interaction of endogenous and exogenous factors plays a crucial role.

Multiple sclerosis has a complex pathophysiology, and numerous risk factors can contribute to its development. It is acknowledged that vitamin D regulates the immune system and helps prevent the onset and spread of autoimmune diseases (Ghareghani et al., 2023). Some recent studies revealed the connection between melatonin levels and immune response and disease activity in MS patients. Melatonin represents a molecule secreted primarily by the pineal gland in response to darkness that has anti-oxidant, anti-inflammatory, and anti-apoptotic capacity (Muñoz-Jurado et al., 2022). Inverse correlations of serum levels of melatonin with the severity of MS, as well as improvement of clinical status once melatonin substitution was introduced, were described recently (Ghareghani et al., 2019). Exposure to sunlight is associated with serum levels of melatonin. Low exposure to sunlight caused by the modern lifestyle with resultant vitamin D insufficiency considerably increases the risk of MS. Epidemiologists have observed an increase in the prevalence of MS in countries with high latitudes, where sunlight is limited and populations suffer from vitamin D insufficiency and high melatonin levels (Ghareghani et al., 2018). Sunlight and UV radiation can influence the immune system in ways other than vitamin D production, such as melatonin production in the pineal gland. Interestingly, systemic inflammatory diseases can inhibit nocturnal melatonin synthesis, altering the circadian rhythm. In MS, a significant increase in serum proinflammatory cytokines and aberrant circulating immune cells may decrease pineal melatonin production (Damasceno et al., 2015). There are significant intraindividual variabilities in melatonin suppression showing a 50-fold difference in sensitivity to light between the least and the most sensitive individuals (Spitschan and Santhi, 2022), with a poor understanding of the causes, some of which include age, sex, chronotypes, and genetic variability (Chellappa, 2020). In psychiatric patients, for example, the suppression of melatonin to light was significantly lower in depressive patients and significantly higher in bipolar disorder, compared to healthy controls (Hallam et al., 2009; McGlashan et al., 2019).

Limited research to date has investigated the volume of the pineal gland in patients with multiple sclerosis. The pineal gland is a small endocrine gland, that has a very important function. It produces melatonin at night, a hormone that regulates sleep-wake cycles and has immunomodulatory properties. Given that MS is an autoimmune disorder characterized by an abnormal immune response (Skarlis and Anagnostouli, 2020), there could be a causative relationship between the size of the pineal gland and MS. A recent study reported on a limited sample size (50 patients), showed that large pineal gland size was related to a lower risk of MS (Kohler et al., 2016). There is not a well-established and widely accepted direct connection between pineal gland volume and multiple sclerosis. Our study is the rare example where this correlation was tested.

Since a positive correlation between pineal gland size and levels of melatonin in sera, saliva, and urine was suggested (Romero-Pinel et al., 2022), in this study we aimed to determine whether the volume of the pineal gland, assessed by magnetic resonance imaging (MRI), correlated with the presence of multiple sclerosis.

## 2 Materials and methods

### 2.1 Subject selection

This retrospective study was conducted on a total of 394 patients, whose magnetic resonance (MR) examinations were available from the database in the period 2008 to 2023. Subjects were divided into two groups: the first group consisted of 188 patients with a definite diagnosis of multiple sclerosis (based on revised McDonald criteria) and the second group consisted of 206 healthy controls. In the first group (MS patients) there were 59 (31.38%) male and 129 (68.62%) female participants, with a mean age of 39.79 ± 12.87 years. In the second group, there were 83 (40.29%) male and 123 (59.71%) female participants with a mean age of 36.48 ± 16.87 years.

To examine the influence of age on pineal gland volume, we divided the whole sample into three age groups: first involved patients under 20 years, second patients between 20 and 40 years, and third group included patients over 40 years. This age threshold was used based on the incidence of multiple sclerosis, which was most common in patients between 20 and 40 years of age (Sigurdardottir et al., 2015).

Inclusion criteria were: subjects of both genders and the presence of MR examination in the hospital database. Exclusion criteria for MS patients were: age under 18, the presence of pineal gland cyst, the presence of brain tumor and metastases, the presence of CNS infection, vascular malformations, active substance abuse and alcohol abuse, and contraindications for MR imaging. Exclusion criteria for healthy controls were: age under 18, the presence of pineal gland cyst, the presence of focal or diffuse lesions in white and gray matter (brain tumors, brain metastases, and congenital anomalies in the brain), the presence of active opportunistic CNS infection, vascular malformations, the history of brain irradiation, active substance abuse, and alcohol abuse, and contraindications for MR imaging.

The study was approved by the institutional ethical review board and the informed consent was waived due to the retrospective manner of the study.

### 2.2 Imaging analysis

Magnetic resonance examinations were performed on two clinical scanners: 1.5T (Siemens Aera, Erlangen, Germany) and 3T (Siemens Trio Tim, Erlangen, Germany). Given that the design of the study was retrospective, the protocols of analyzed examinations were not identical. All the patients had conventional MR examination consisting of T1W sagittal, T2W, FLAIR, DWI axial, and T2W coronal images.

**FIGURE 1:**
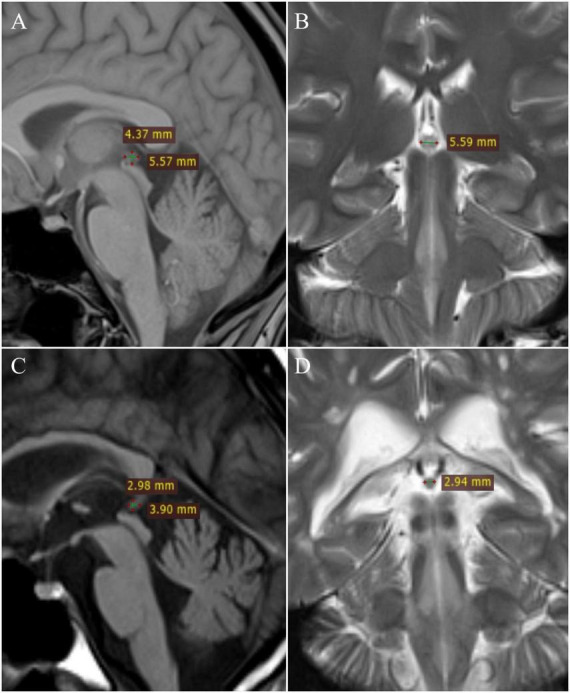
Measurement of pineal gland: healthy patient – **(A)** T1W sagittal and **(B)** T2W coronal; multiple sclerosis patient – **(C)** T1W sagittal and **(D)** T2W coronal.

All images were analyzed on the workstation (Leonardo) by two independent readers in consensus. The maximum length (L) and height (H) of the pineal gland were measured on the T1-weighted sagittal images, and the width (W) was measured on the T2-weighted coronal or axial images, according to Sumida et al. (1996). The volume of the gland was calculated as an approximation to an ellipse, according to the formula *V* = (*L* × *H* × *W*)/2 (Figure 1).

### 2.3 Statistical analysis

Statistical analysis was performed and calculated using the SPSS v27.0 software tool (SPSS Inc., IBM, Armonk, NY, USA). The confidence interval was set to 95%, with a significance level of *p* < 0.05.

Differences in the pineal gland volume and age of the participants between study groups, both males and females, were analyzed with the Mann–Whitney U test because the data were continuous with abnormal distribution which was examined with the Kolmogorov–Smirnov test. Also, the Mann–Whitney U test was used to check differences between males and females both in patients with multiple sclerosis and healthy controls. Analysis of pineal gland volume difference between age groups in both study groups was examined with the Kruskal–Wallis test. Pearson correlation coefficient (*r*) was used to investigate the correlation between age and pineal gland volume in both study groups and among both sexes, to isolate the influence of the disease on the pineal gland volume. For pineal gland volume, the 25th, 50th, and 75th percentile were obtained and among quartiles, each odds ratio was determined between patients with multiple sclerosis and healthy controls.

## 3 Results

### 3.1 Demographic data

The total number of subjects included in this research was 394, of which 188 were patients with multiple sclerosis and 206 were healthy controls. In the group of patients with multiple sclerosis, 59 (31.38%) men and 129 (68.62%) women were examined, with a mean age of 39.79 ± 12.87 (range 18–69) years, while the healthy control group included 83 (40.29%) men and 123 (59.71%) women, with the mean age of 36.48 ± 16.87 (range 18–70) years. The sample was coherent with regards to age and gender – the data is detailed in Tables 1, 2.

**TABLE 1:** Demographic data for patients with multiple sclerosis and healthy controls.

Groups	*N* (%)	Age ($\overset{¯}{X}$ ± SD)
Multiple sclerosis	188	39.79 ± 12.87
Male	59 (31.38)	39.47 ± 13.70
Female	129 (68.62)	39.94 ± 12.53
Healthy controls	206	36.48 ± 16.87
Male	83 (40.29)	34.55 ± 15.98
Female	123 (59.71)	37.78 ± 15.23

**TABLE 2:** Sex differences in pineal gland volume (mm^3^) between groups.

Sex	Multiple sclerosis	Healthy controls	*p*-Value
Male	47.57 ± 22.16	52.82 ± 26.44	0.207
Female	45.06 ± 22.58	52.90 ± 25.13	0.013
Total	45.84 ± 22.42	52.87 ± 25.60	0.005

### 3.2 Differences in pineal gland volume between multiple sclerosis patients and healthy controls

**FIGURE 2:**
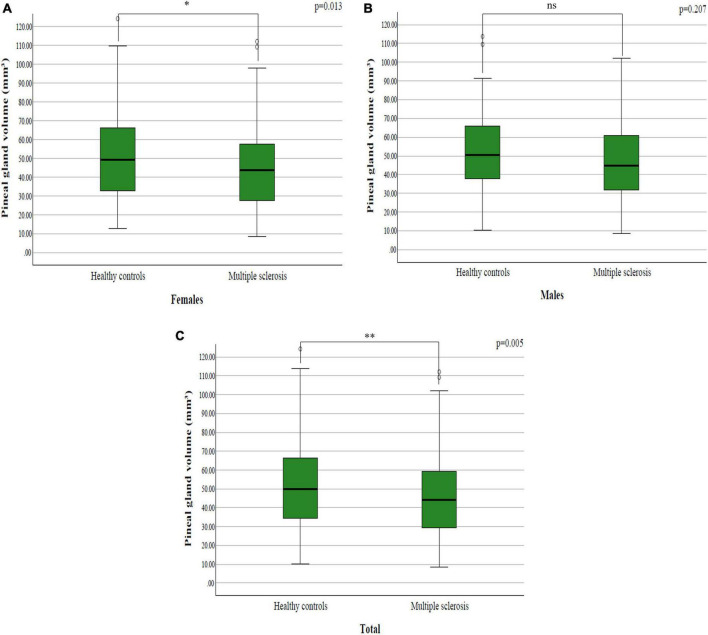
The difference in pineal gland volume between patients with multiple sclerosis and healthy controls among: **(A)** females; **(B)** males; **(C)** total sample.

By examining pineal gland volume between the study groups, it was determined that they differ significantly both in the whole group (*p* = 0.005; *p* < 0.05) (Figure 2C) and among females (*p* = 0.013; *p* < 0.05) (Figure 2A), while in males there was no significant difference (*p* = 0.207; *p* > 0.05) (Figure 2B and Table 2).

### 3.3 Differences in pineal gland volume between females and males in healthy controls

**FIGURE 3:**
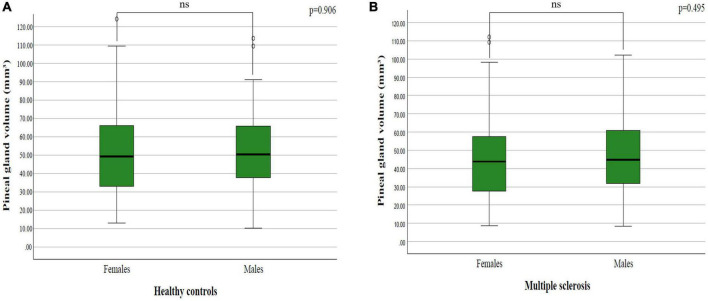
The difference in pineal gland volume between females and males in: **(A)** healthy controls and **(B)** multiple sclerosis patients.

The mean pineal gland volume in females in healthy controls was 52.90 ± 25.13 mm^3^, while in males it was 52.82 ± 26.44 mm^3^. Data processing did not establish a statistically significant difference between sexes in healthy controls (*p* = 0.906; *p* > 0.05) (Figure 3A).

### 3.4 Differences in pineal gland volume between females and males in patients with multiple sclerosis

The mean pineal gland volume in females in patients with multiple sclerosis was 45.06 ± 22.58 cm^3^, while in males it was 47.57 ± 22.16 mm^3^. By analyzing the difference between sexes in patients with multiple sclerosis, we did not find a statistically significant difference (*p* = 0.495; *p* > 0.05) (Figure 3B).

### 3.5 Differences in pineal gland volume between various age groups among patients with multiple sclerosis and healthy controls

Both study groups, multiple sclerosis and healthy control patients, were further subdivided into groups depending on age to find out the influence of age on pineal gland volume. Three age groups were: under 20 years, between 20 and 40 years, and over 40 years of age. There was not any statistically significant difference in the pineal gland volume depending on the age group in both males and females, neither in patients with multiple sclerosis nor healthy controls (Table 3).

**TABLE 3:** Pineal gland volume in various age groups.

Groups	<20 years	≥20 and ≤40 years	>40 years	*p*-Value
Multiple sclerosis	43.27 ± 24.59	47.91 ± 24.99	44.30 ± 19.83	0.779
Male	46.61 ± 32.63	47.91 ± 25.51	47.40 ± 16.76	0.888
Female	39.10 ± 12.29	47.90 ± 24.97	43.09 ± 20.89	0.585
Healthy controls	52.55 ± 31.05	56.15 ± 25.87	50.54 ± 22.47	0.260
Male	58.98 ± 38.40	57.48 ± 21.20	44.21 ± 14.82	0.050
Female	43.62 ± 12.42	55.37 ± 28.48	53.86 ± 25.06	0.357

We also analyzed if there is a statistically significant difference in pineal gland volume when comparing same age groups among and between patients with multiple sclerosis and healthy controls. The only two groups that showed significant difference were healthy subjects over 40 years of age, where males had lower volume of the gland (*p* = 0.049; *p* > 0.05; *t*-test), and a second group that showed difference were females with multiple sclerosis over 40 years of age who had lower pineal gland volume in comparison to healthy females over 40 years of age (*p* = 0.024; *p* < 0.05; Mann–Whitney U test).

### 3.6 Correlation between age and pineal gland volume in study groups

We wanted to determine if there is any correlation between age and pineal gland volume in study groups, both in males and females, to isolate the potential influence of disease on gland volume. Analyzing data, we did not find any correlation between age and gland volume neither in healthy controls nor patients with multiple sclerosis (Table 4).

**TABLE 4:** Correlation between age and pineal gland volume in groups.

Groups	Pearson correlation coefficient (*r*)	*p*-Value
Multiple sclerosis	−0.020	0.783
Male	0.130	0.325
Female	−0.092	0.299
Healthy controls	−0.048	0.493
Male	−0.194	0.078
Female	0.080	0.380

### 3.7 Risk of multiple sclerosis in reference to pineal gland volume

Regarding pineal gland volume, values for the 25th, 50th, and 75th percentile were 31.85, 45.75, and 62.83 mm^3^. In categorical analyses using the lowest quartile as a reference, the odds ratio for each subsequent quartile was 0.71, 0.74, and 0.42 (*p* = 0.026 for trend across quartiles). In addition, the odds ratio from the highest quartile was significantly different from 1 (odds ratio 0.42; 95% confidence interval 0.23–0.74; *p* = 0.003). Our results suggest that large pineal gland volume, especially over 62.83 mm^3^ when compared to pineal gland volume below 31.85 mm^3^ is associated with more than double reduced risk of multiple sclerosis.

## 4 Discussion

Melatonin is a potent antioxidant, anti-inflammatory, and antinociceptive that also enhances mitochondrial function, possibly through increased deoxidative phosphorylation. Melatonin also suppresses demyelination while increasing remyelination. Melatonin’s involvement in MS, however, may be dependent on local glial melatonin production and release, rather than pineal-derived melatonin (Anderson and Rodriguez, 2015). Patterns of vitamin D and melatonin synthesis are opposite. Vitamin D is produced by the skin during daylight/ exposure to the sunshine while melatonin is produced by the pineal gland during the night/ in the absence of any light – meaning that stimulation of the one’s production will suppress the production of another agent. The study by Golan et al. (2013) showed that 1-year daily treatment of MS patients with high dosage vitamin D significantly suppressed nighttime secretion of melatonin, suggesting mediation of vitamin D effect via melatonin in MS.

There are four types of MS: progressive relapsing, primary-progressive (PPMS), relapsing-remitting (RRMS), and secondary-progressive form (Compston and Coles, 2008). Recent studies showed not only the decrease in nocturnal secretion of melatonin but also the correlations with the disease stage (Álvarez-Sánchez et al., 2017). The first associations between melatonin level and the course of MS were established in the early 1990s suggesting that progressive decline in melatonin secretion can be a function of MS duration: with the progression of the disease there is a steady decline in pineal gland function and the immunosuppressant effect wanes thus resulting in the poorer ability to recover from exacerbations (Sandyk and Awerbuch, 1994). This early hypothesis was further confirmed with lower mean melatonin levels observed in patients with chronic progressive MS compared to those with relapsing-remitting course.

The only study found in the literature that investigated the association of pineal gland volume and MS is from Kohler et al. (2016), which included 50 MS patients. Their study results suggest that large pineal gland size is associated with a lower risk of multiple sclerosis which is in accordance with our results. Unlike their study, we analyzed 188 MS patients and also compared the pineal gland volume of MS patients with healthy controls and the results showed significantly lower pineal gland in MS patients in the female sample, whereas in males we have not found a significant difference. The reason for that may be due to fewer male patients involved in the study. However, it is well-established that MS is a female-predominant disease with only about 25% of patients being males (Coyle, 2021). The additional important difference is the different type of MS that is predominant in females – the RRMS, while in men the most predominant type is the PPMS (Safi and Krieger, 2021). When looking at the sex differences, the pineal gland volume did not differ between males and females, both in MS patients and healthy controls.

In an attempt to exclude any potential cause of age-affected influence on pineal gland volume in both MS patients and healthy controls, we analyzed the Pearson correlation coefficient and did not find a correlation between age and pineal gland volume in any group. That provides us with an important information that the pineal gland volume is not age-dependent, and the observed smaller pineal gland in MS patients can reliably be attributed to the disease itself.

A potential explanation of our study results lies in the fact that the pineal gland is connected through neural paths with the eyes and melatonin produce is suppressed by the light stimulus (Sandyk, 1997). The incidence of MS is higher in geographical places with shorter daylight (Ghareghani et al., 2023). This can in part potentially be explained by the suppression of melatonin produced by the excessive (artificial) light stimuli during the night. Melatonin levels have recently been linked to the severity and potential of MS to relapse (Ghareghani et al., 2018). Additionally, current studies showed a beneficial effect of melatonin as a supplement in relieving some of the MS symptoms and improving the quality of life (Skarlis and Anagnostouli, 2020). Moreover, the research is being conducted on melatonin as a therapeutic option in the design of therapy regimens in MS (Ghareghani et al., 2023). Pineal volume can reflect the production of melatonin, given that ca. 80% of the gland is composed of functional, secreting cells – pinealocytes (Sigurdardottir et al., 2015). Even though there are large individual differences in the amount of melatonin secretion (Arendt, 2006), the volume of the solid part of the pineal gland was positively correlated with saliva melatonin levels collected at 4-time points during 24h (Liebrich et al., 2014) as well as with 24h melatonin levels measured in the 2h intervals (Nolte et al., 2009). However, the exact relationship between pineal gland volume and MS is not yet fully understood and requires further research. Additionally, reduction of pineal gland volume has been detected in various disorders like schizophrenia (Takahashi et al., 2019), Alzheimer’s dementia (Matsuoka et al., 2018), mild cognitive impairment (Matsuoka et al., 2020), and sleep disorders (Park et al., 2020), which reflect its importance.

This study has some limitations. A major limitation of the study is the retrospective manner that resulted in the lack of detailed clinical data, on the levels of melatonin in the first place, which might contribute to a better understanding of the role of pineal gland diameters and volume in the pathogenesis of MS or its prognosis.

Another limitation of the study was the absence of a unique MR imaging protocol (some examinations lacked 1 mm thick slices) that would certainly improve the accuracy of performed measurements. Finally, the approximation of the pineal gland shape to an ellipse was used for volume assessment (Sumida et al., 1996) instead of volumetry or planimetry. Studies that are more recent used planimetry and point-counting methods and found no significant differences between measurements obtained on various methods (Acer et al., 2012). The method we used in our opinion, is clinically useful and can be used in everyday routine practice, given that volumetric analyses (if not automated) tend to be time-consuming.

Finally, the data were collected in a retrospective manner and in the setting of an outpatient clinic covering several centers for MS pathology in the state; this prevented us from collecting reliable data on MS type, stage and duration of the disease, so the exact causative relationship between pineal gland volume loss and MS cannot be reliably established.

Our results suggest that women with multiple sclerosis have smaller pineal glands that can theoretically be explained by a lack of input stimuli and the resultant decrease in gland volume. Additionally, the risk of multiple sclerosis is reduced in larger pineal gland volumes. Further endocrine and metabolic studies are needed in the future to explain the decrease in pineal gland volume in women with multiple sclerosis.

## Data availability statement

The raw data supporting the conclusions of this article will be made available by the authors, without undue reservation.

## Ethics statement

The studies involving humans were approved by the Oncology Institute of Vojvodina. The studies were conducted in accordance with the local legislation and institutional requirements. The ethics committee/institutional review board waived the requirement of written informed consent for participation from the participants or the participants’ legal guardians/next of kin since this is a retrospective study.

## Author contributions

MV: Writing – original draft, Writing – review & editing. IN: Writing – original draft, Writing – review & editing. JB: Formal analysis, Supervision, Writing – original draft, Writing – review & editing. DK: Conceptualization, Formal analysis, Investigation, Supervision, Validation, Visualization, Writing – original draft, Writing – review & editing.
